# Region-specific alterations of A-to-I RNA editing of serotonin 2c receptor in the cortex of suicides with major depression

**DOI:** 10.1038/tp.2016.121

**Published:** 2016-08-30

**Authors:** D Weissmann, S van der Laan, M D Underwood, N Salvetat, L Cavarec, L Vincent, F Molina, J J Mann, V Arango, J F Pujol

**Affiliations:** 1 ALCEDIAG/CNRS Sys2Diag FRE3690, Biological Complex System Modeling and Engineering for Diagnosis, Cap delta/Parc Euromédecine, Montpellier, France; 2 Division of Molecular Imaging and Neuropathology, Columbia University College of Physicians and Surgeons, New York, NY, USA; 3 New York State Psychiatric Institute, New York, NY, USA

## Abstract

Brain region-specific abnormalities in serotonergic transmission appear to underlie suicidal behavior. Alterations of RNA editing on the serotonin receptor 2C (HTR2C) pre-mRNA in the brain of suicides produce transcripts that attenuate 5-HT_2C_R signaling by impairing intracellular G-protein coupling and subsequent intracellular signal transduction. In brain, the distribution of RNA-editing enzymes catalyzing deamination (A-to-I modification) shows regional variation, including within the cerebral cortex. We tested the hypothesis that altered pre-mRNA 5-HT_2C_R receptor editing in suicide is region-specific. To this end, we investigated the complete 5-HT_2C_R mRNA-editing profile in two architectonically distinct cortical areas involved in mood regulation and decision-making in a clinically well-characterized cohort of age- and sex-matched non-psychiatric drug-free controls and depressed suicides. By using an original biochemical detection method, that is, capillary electrophoresis single-stranded conformational polymorphism (CE-SSCP), we corroborated the 5-HT_2C_R mRNA-editing profile previously described in the dorsolateral prefrontal cortex (Brodmann area 9 (BA9)). Editing of 5-HT_2C_R mRNA displayed clear regional difference when comparing dorsolateral prefrontal cortex (BA9) and anterior cingulate cortex (BA24). Compared with non-psychiatric control individuals, alterations of editing levels of 5-HT2CR mRNA were detected in both cortical areas of depressed suicides. A marked increase in editing on 5-HT_2C_R was especially observed in the anterior cingulate cortex in suicides, implicating this cortical area in suicide risk. The results suggest that region-specific changes in RNA editing of 5-HT_2C_R mRNA and deficient receptor function likely contribute to the etiology of major depressive disorder or suicide.

## Introduction

Suicide and suicidal behavior are major public health concerns worldwide. According to a very recent report, over a million suicides are reported per year worldwide.^1^ A main risk factor for suicide is a psychiatric illness.^2, 3^ Suicide is a complex multifactorial outcome and its biological basis remains insufficiently understood. Although neither a single gene nor a single signaling pathway may entirely account for the development of a complex disease, low serotonergic activity and brain regional abnormalities in serotonin neurotransmission have been proposed as biological traits related to suicidal behavior.^4^ Among the serotonin receptor family, the serotonin 2C receptor (5-HT_2C_R) is of particular interest, as it has been associated with regulation of mood, appetite, sleep and sexual behavior.^5, 6^ The serotonin 2C receptor is heterogeneously distributed in the brain, and, aside from the choroid plexus, is more abundant in the hypothalamus, hippocampus, prefrontal cortex and in regions containing dopamine and serotonin-synthesizing neurons.^7^ Very recently a study identified two polymorphisms located in, respectively, the ADAR2 (RNA deaminase acting on RNA) and HTR2C genes associated with suicidal attempts, linking genetic and epigenetic factors to elevated risk of suicide.^8^

Adenosine deaminase acting on RNA (ADAR) enzymes target double-stranded pre-mRNAs' stem loops to specifically deaminate preferential adenosine residues (called A-to-I editing of RNA). Analysis of the spatiotemporal expression in mouse forebrain of ADAR1 and ADAR2 enzymes revealed developmental control and high transcript levels across nearly all brain regions in adulthood. Co-labeling studies further showed that ADAR1- and ADAR2-immunoreactive cells stained positive for neuronal nuclei and negative for GFAP, indicating that both ADAR enzymes specifically are expressed in neurons and not in most glial cells within the mouse cerebral cortex and hippocampus.^9^ Overall distribution of ADAR1 and ADAR2 in the adult brain was comparable, although considerable regional specificities for each enzyme were observed.^9^ For still enigmatic reasons, A-to-I editing of RNA essentially seems to bring about protein recoding in highly conserved neurotransmitters and synapse-related factors, supposedly reflecting its key role in neurobiology^10, 11, 12^ and in modulation of neurotransmission.^13^

In case of the serotonin 2c receptor, deamination affects the amino-acid composition of the second intracellular loop of the 5-HT_2C_R receptor, inducing conformational changes that all decrease G-protein-coupling activity, agonist affinity and thus serotonin signaling.^14, 15, 16, 17^ On the 5-HT_2C_R receptor pre-mRNA, five adenosine residues contained within 20 bases located on exon V are targeted by the ADAR enzymes and termed A to E sites. Editing at A, B and C sites is ADAR1-specific, whereas editing at the D site is attributed to ADAR2 enzymatic activity.^18^ The E site (previously coined as C′) is both edited by ADAR1 or ADAR2 enzymes. A-to-I RNA editing can result in up to 24 different receptor isoforms, each with specific activity. In addition, RNA editing also affects differential splicing and intracellular trafficking of 5-HT_2C_R receptor,^19, 20^ which can also alter receptor function. Differences in the relative proportions of these isoforms appear to be linked to the biology of suicide as it could link deficient serotonin function to variations in RNA editing of the 5-HT_2C_R receptor. This is in line with proposed attenuated serotonin neurotransmission and 5-HT_2C_R receptor function reported in suicide.^14, 21, 22, 23^ Moreover, post-mortem analyses of the dorsolateral prefrontal cortex (DLPFCx) of 5-HT_2C_R mRNA-editing profiles in suicides with psychiatric disorders as major depressive disorder (MDD), schizophrenia and bipolar disorder consistently showed increased levels of these epigenetic modifications in suicides regardless of the underlying disease.^24, 25, 26, 27^

**Figure 1:**
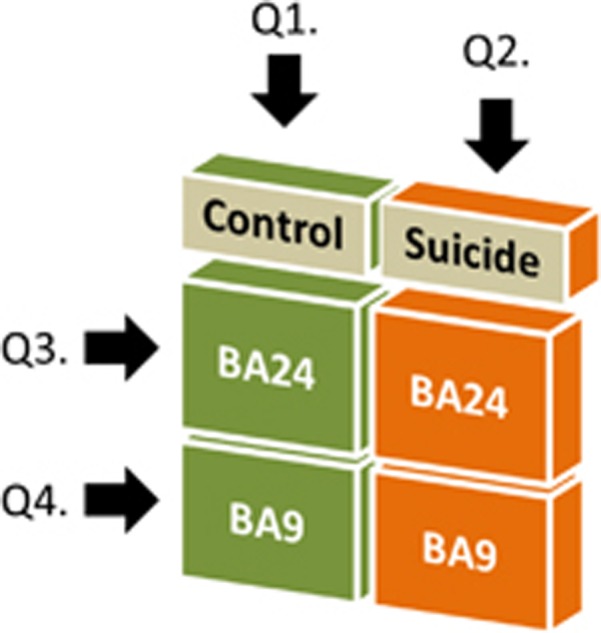
Schematic representation of the different analyses performed, questions addressed and baseline characteristics of the population. Q1: Do brain region-specific differences in 5-HT_2C_R mRNA editing in control individuals exist? Q2: How does suicide influence 5-HT_2C_R mRNA editing in two distinct cortical areas? Q3–4: Is suicide having an impact on editing of 5-HT_2C_R mRNA in Brodmann area 9 (BA9; Q3) and BA24 (Q4)?

In the current study, we take advantage of a novel technology, validated in the mouse brain,^28^ to examine the complete editing profile of 5-HT_2C_R mRNA in two architectonically distinct neocortical regions in non-psychiatric, drug-free accidental controls and depressed suicides matched for age and sex. We compared 5-HT_2C_R mRNA editing in Brodmann areas 9 (DLPFCx) and 24 (anterior cingulate cortex (ACCx)), both known to be critically involved in mood regulation and cognitive control processes.^29^ Alterations in the DLPFCx have been reported in mood disorders, both *in vivo*^30, 31^ and postmortem.^32^ The ACCx is a major component of the ‘limbic system' and is implicated in emotion, attention, mood states, motor and cognitive processes.^33, 34, 35^ We first investigated whether differences could be found in 5-HT_2C_R mRNA editing between Brodmann area 9 (BA9) and BA24 within non-psychiatric controls and suicides (Figure 1; Q1–2). Next, we analyzed both brain regions independently to identify specific suicide-related 5-HT_2C_R mRNA alterations (Figure 1; Q3–4).

## Materials and methods

### Subjects

Study procedures were approved by the applicable institutional review boards. Consent was given by next-of-kin for tissue collection, review of relevant records and interviews for a psychological autopsy. Tissue was provided by the Conte Center for the Neurobiology of Mental Disorders, Human Neurobiology Core Brain collection, at the New York State Psychiatric Institute. All cases died suddenly (see Table 1 for demographic and clinical details). Brains were collected at autopsy. The mesencephalon was detached from the diencephalon and the forebrain was bisected. Samples from the left cerebral hemisphere were placed in fixative for neuropathological examination. The right hemisphere was sectioned coronally into ~11 blocks, each placed on a glass slide, carefully immersed in Freon R-12 for flash-freezing, stored in sealed thick plastic bags and kept at −80 °C until dissection.^36, 37, 38^ Body fluids and brain tissue underwent extensive toxicological screening. Individuals with a history of cerebral trauma, central nervous system disease, chronic alcoholism, illicit or therapeutic drug use or AIDS were excluded. Body fluids (blood, bile, aqueous humor and urine) were used for toxicological screening for cocaine, opiates, alcohol, antidepressants and other acidic and basic drugs. All cases underwent brain toxicological screens as well and, except for case number 15, which was positive for fluoxetine, all cases were negative for medication known to affect serotonin (Supplementary Table S1). The brain samples were coded and assayed by a personnel blind to the cause of death. In order to assess the integrity of the tissue RNA, we measured pH in the cerebellum as described by Harrison *et al.* (Table 2).^39^ At least one informant per case agreed to an interview for the purpose of a psychological autopsy, which was performed according to our previously reported method.^40^ A psychological autopsy was used to obtain DSM-IV Axis I and II diagnoses using the SCID I and II,^41, 42^ as we previously validated.^40^ Further details about the psychological autopsy procedure can be found elsewhere.^37, 38, 43^ Control subjects (*n*=8), who died from causes other than suicide, did not meet criteria for any Axis I diagnosis during their lifetime. Suicides (*n*=8) met criteria of the Columbia Classification of suicidal behavior (Table 1).^44^ History of prior suicide attempts was determined using the Columbia Suicide History Form.^45^ All the subjects in the suicide group met criteria for MDD at least once during their lifetime and did not meet criteria for bipolar disorder or psychotic disorders. Case number 16 met criteria for gambling disorder and obsessive compulsive disorder (Supplementary Table S1).

### Brain regions

The DLPFCx and the ACCx were selected because they have been consistently implicated and altered in depression and/or suicide.^46^

### DLPFCx (Brodmann Area 9)

We dissected a sample of BA9 from a frozen block using the delineations by Petrides and Pandya.^47^ Sections (50μm) were taken from a frozen hemispheric coronal block at a level anterior to the genu of the corpus callosum and stained for Nissl (Supplementary Figure S1A, B). We identified BA9 by its location and the lack of a well-developed granular Layer IV, clearly distinguished from the adjacent BA46 that has a well-developed granular Layer IV. Using the Nissl-stained section as a guide (Supplementary Figure S1B), tissue was punched for subsequent RNA extraction.

### ACCx (Brodmann Area 24)

We dissected BA24 from a frozen block just posterior to the genu of the corpus callosum (Supplementary Figure S1C). A section was stained for neuron-specific nuclear protein (neuronal nuclei) in order to visualize cytoarchitectonic features (Supplementary Figure S1D), and we took a punch from the dorsal ACCx, which is proisocortical agranular cortex, characterized cytoarchitectonically by the absence of a Layer IV in human.^48^

### RNA isolation, complementary DNA synthesis and PCR

Total RNA was extracted from brain specimens and was controlled for integrity^49^ (Supplementary Figure S2). Total RNA was extracted from brain specimens, purified (Qiagen RNeasy, Kit; Qiagen, Hilden, Germany), quantified by spectrophotometry, treated with 1 unit of DNase I (Invitrogen, Carlsbad, CA, USA) for 15 min at room temperature in a final volume of 10 μl, then 1 μl of 25 mM EDTA was added and the mixture heated for 10 min at 65°C. Next, total RNA was qualified by electrophoresis and the RNA integrity number (RIN) score was determined for each total RNA sample (Supplementary Figure S2). Reverse transcription was performed using 15 units of ThermoScript reverse transcriptase (ThermoScript RT-PCR System, Invitrogen) in presence of Oligo(dT) primers at a final concentration of 2.5 μM. Prior analysis of 5-HT2CR receptor RNA editing levels, non-denaturating capillary electrophoresis-single stranded conformational polymorphism (CE-SSCP) procedure was calibrated using plasmid containing exactly a 250bp long cDNA sequence coding respectively for all 32 possible edited isoforms (Supplementary Figures S3 and S4). All expression plasmids were verified by DNA sequencing. An initial amplification by PCR (final volume 25 μl) was carried out on 1 μl of the obtained cDNA using 0.2 unit of Platinum Taq DNA polymerase (ThermoScript RT-PCR system, Invitrogen) and specific intron-spanning 5-HT2CR primers (forward primer: 5′-TGTCCCTAGCCATTGCTGATATGC-3′ and reverse primer: 5′-GCAATCTTCATGATGGCCTTAGTC-3′ final concentration of each 0.2 μM) located on exon IV and exon V, respectively. The PCR protocol consisted of an initial denaturing step at 95°C for 3 min, 35 cycles of amplification (15s at 95°C; 30s at 60°C; 20s at 72°C), and a final elongation step of 2 min at 72°C. One μl of a 1/50 dilution of the PCR products or the 250 bp cDNA amplified from plasmids containing the 32 standards of human 5-HT2CR R isoforms, were used as templates for nested-PCR. The second amplification was performed in a final volume of 20 μl with VIC and FAM fluorescent primers. The primer sequences used were: Forward: 5′-ATGTGCTATTTTCAACAGCGTCCATC-3′ and Reverse: 5′-GCAATCTTCATGATGGCCTTA-3′. This set of primer pair was optimised for conformational analysis of human 5-HT2CR mRNA editing by non-denaturing CE-SSCP. The length of the 5 amplified fragment was carefully chosen (127 bp). Quality of the amplified fragments was assessed on a 2% agarose gel before subsequent analysis in a 3100 Avant Genetic Analyser (Applied Biosystems, Courtaboeuf, France).

### Identification and relative quantification of RNA editing by CE-SSCP

Fluorescent PCR products obtained following the second amplification and corresponding to standard isoforms and samples were added to a mixture of ROX-labeled migration standards (MWG-BIOTECH, AG; 0.5 μl each) covering the whole range of the retention times required for CE single-stranded conformational polymorphism (CE-SSCP) analysis. After denaturing for 2 min at 95 °C, the samples were immediately chilled on ice (Supplementary Figure S3 for schematic overview of the procedure). Non-denaturing CE was carried out in an ABI PRISM 3100-*Avant* Genetic Analyser (Applied Biosystems) through 80 cm-long capillaries filled with 7% ‘POP Conformational Analysis Polymer' (Applied Biosystems), 1 × TBE and without glycerol.

After a pre-run performed at 15 kV for 3 min, the samples were injected for 15s at 2 kV, and electrophoresis was run for 105 min at 15 kV at a controlled temperature of 20 °C. Under these conditions, the separation of the 32 possible isoforms was resolved. The electrophoretic signal was then processed using a software, allowing deconvolution of the isoform standards and sample signals in a unique time basis. Background was adjusted, subtracted and the total area under each signal normalized. The relative proportion of each isoform was processed by a best fitting of each deconvoluted and normalized analytical signal of the brain sample (Supplementary Figure S4). It was performed by the iterative adjustment of the integrated signal represented by the 32 similarly processed signals of the standards and was controlled by the least squares statistical analysis. A relative proportion of at least 0.5% was set as the threshold in order to be included in the analysis. All experiments were carried out under masked conditions and all samples from the two brain regions were assayed in the same batch for complementary DNA synthesis and PCR amplifications.

### Statistical analysis and design of the analysis

Statistical analyses and figures were generated using the ‘R/Bioconductor' statistical open source software (version 3.2.0).^50^ Differences between groups were analyzed by using the non-parametric Wilcoxon rank-sum test. *P*-values <0.05 were considered as statistically significant. Significant 5-HT_2C_R editing isoform distributions are illustrated with boxplots and medians. The calculation of the relative 5-HT_2C_R isoform proportion takes into account all possible deviations between two sets of experimental conditions and is defined through the following formula:


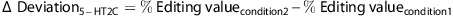


The following two criteria were considered for further detailed analysis: variations of the median value of relative proportion of RNA editing above 20% and *P*-values <0.05 using the one-sample Wilcoxon rank-sum test (where null hypothesis H0: median variation of % editing value=0).

The data quality evaluation was performed by three distinct approaches: (1) dispersion trees, (2) Pearson correlation matrix and (3) principal component analysis. (A detailed description of these methods can be found in Supplementary Materials and Methods.) On the basis of the quality assessment of the RNA-editing data, three samples showing clear deviations with respect to the other subjects were excluded from our analysis (Supplementary Figure S5). Quality-assessment analysis was reiterated and exclusion of the three individuals resulted in a highly homogenous data set (Supplementary Figure S5B). Of note, analysis of both the complete and the filtered data sets resulted in similar findings.

## Results

### Subjects

A paired case–control design was used to control demographic and assay variance. Potential confounding factors such as age, ethnicity of subjects, post-mortem interval, extracted tissue weight or tissue pH were not statistically different between control and MMD groups (Tables 1 and 2). In order to avoid potential sex differences, only male subjects were studied. Raw data of the complete RNA-editing profiles are shown in Supplementary Figures S6, S7.

### Full-profile analysis of 5-HT_2C_R editing by CE-SSCP

**Figure 2:**
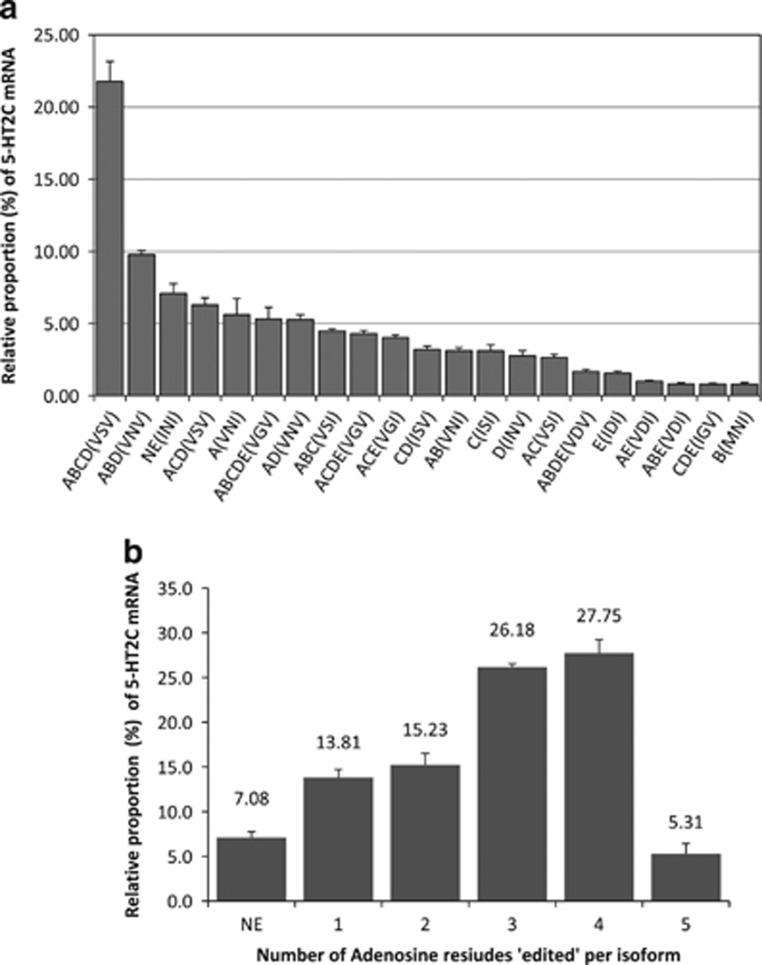
Relative isoform proportion of 5-HT_2C_R mRNA in Brodmann area 9 (BA9) measured by capillary electrophoresis single-stranded conformational polymorphism (CE-SSCP) on samples of the control group. (**a**) Histograms represent relative isoform proportion (%) of the 21 foremost detected 5-HT_2C_R isoform (means±s.e.m.; *n*=7). Only isoforms representing more than 0.5% of relative proportion were included in the analysis. (**b**) Relative distribution of 5-HT_2C_R mRNA isoforms grouped by the number of ‘edited' adenosine sites within the studied sequence. Histograms represent the cumulative relative proportion of all isoforms. The complete data set of 5-HT_2C_R mRNA editing in BA9 and BA24 in control and suicide groups can be found in Supplementary Figure S7.

In the non-psychiatric control group, the mean cumulative relative proportion of isoforms representing less than 0.5% of the 5-HT_2C_R mRNA did not exceed 5% and was found to be higher in BA9 (4.6%) compared with BA24 (2.4% Supplementary Figures S6 and S7). In BA9, the relative proportion of the non-edited (NE) isoform was 7.1, as 92.9% of the isoforms were identified as being edited at least on one site. The relative proportion of the edited isoform ABCD was the highest and represented 21.8% of all 5-HT_2C_R mRNAs analyzed (Figure 2a). The fully edited (ABCDE) isoform was the sixth most prevalent isoform with a relative proportion of 5.3%. Further analysis of the isoforms in terms of number of edited sites revealed that the largest proportion of 5-HT_2C_R had four edited adenosine residues, with a cumulative fraction of ABCD, ACDE and ABDE isoforms representing 27.8% of the total mRNA (Figure 2b). The relative proportion of isoforms bearing three or four edited adenosines represented almost 55% of the total 5-HT_2C_R isoforms. Taken together, the current RNA-editing profile of 5-HT_2C_R mRNA corroborates previous data^26^ and is indicative of a high RNA-editing activity mediated by ADAR enzymes in the DLPFCx.

### Brain regional differences in 5-HT_2C_R mRNA-isoform proportion

**Figure 3:**
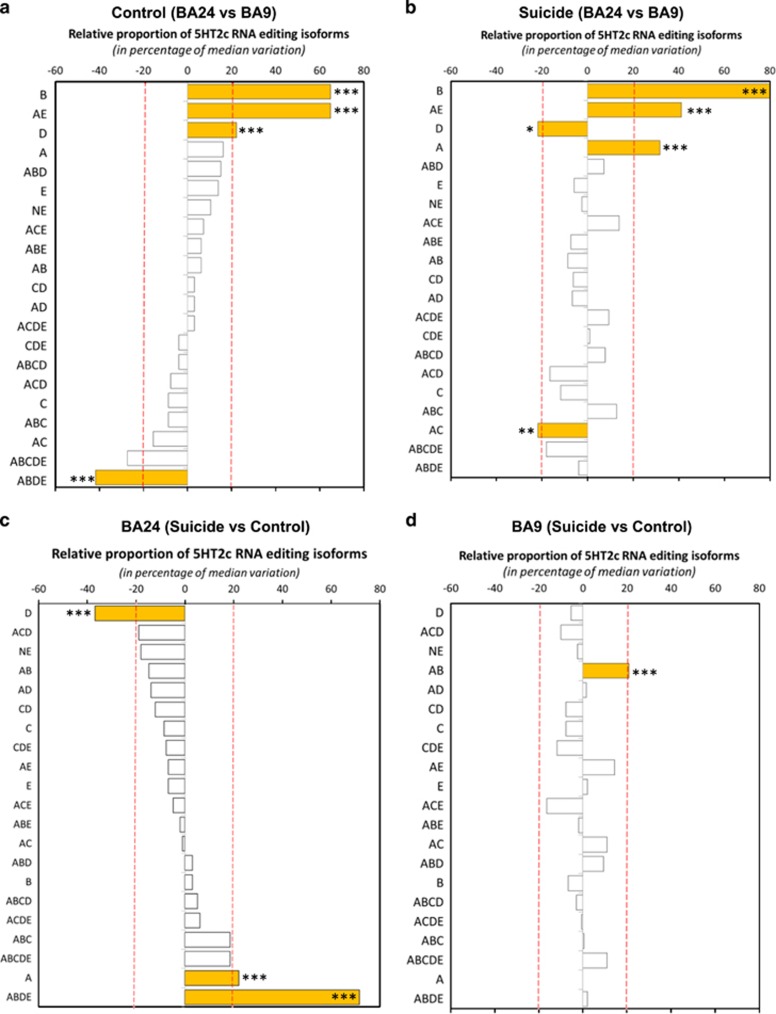
Brain regional specificity of changes in 5-HT_2C_R mRNA editing in suicide victims and non-psychiatric controls. (**a**) Comparison of the relative isoform proportion of 5-HT_2C_R mRNA in BA24 (*n*=7) and Brodmann area 9 (BA9; *n*=7) within the control group (see Figure 1a Q1). The 5-HT_2C_R isoforms are depicted in order of statistical significance and relative proportion in BA24. (**b**) Comparison of the relative mRNA-isoform proportion of 5-HT_2C_R in BA24 (*n*=7) and BA9 (*n*=8) in the suicide group in the same order as in **a** (see Figure 1a Q2). Filled yellow bars indicate the most significant differences in relative proportion of the isoform between the two brain structures in both control and suicide groups. (**c**) Comparison of the relative isoform proportion of 5-HT_2C_R in control (*n*=7) and suicide (*n*=7) groups within BA24 area (see Figure 1a Q3). The 5-HT_2C_R isoforms are depicted in order of statistical significance and relative proportion in the suicide group. A negative value indicates a relative decrease in isoform presence. Inversely, an increase in the relative proportion is indicated by a positive value. (**d**) Comparison of the relative isoform proportion of 5-HT_2C_R in control (*n*=7) and suicide (*n*=8) groups within the BA9 area (see Figure 1a Q4). Filled yellow bars indicate most significant differences between control and suicide groups. Criteria for the selection are (1) a *P*-value ⩽0.05 using the one-sample Wilcoxon signed rank test (where null hypothesis H0: median variation=0) and (2) a median variation less than equal to ±20%. *P⩽0.05, **P⩽0.01 and ***P⩽0.001.

When comparing the relative proportion of 5-HT_2C_R mRNA isoforms in BA24 to BA9 in non-psychiatric control subjects (Figure 3a), 4 of the 21 isoforms displayed a difference greater than 20% from the median between the two cortical regions. In our representation, a negative percentage from the median indicates that the relative proportion of the isoform is higher in BA9 compared with BA24. In non-psychiatric control subjects, the relative proportion of B and AE isoforms was 65% higher (*P*<0.001) and that of D was 22% higher in BA24 compared with BA9 (Figure 3a). Conversely, the ABDE isoform, which requires both ADAR1- and ADAR2-editing activities, was more frequent in BA9 (−42% from the median value, *P*<0.001). The most-edited isoform (ABCDE) had an overall median value of 27% higher in BA9 compared with BA24, but with a *P*-value >0.05 (Figure 3a). When comparing the relative proportion of 5-HT_2C_R mRNA isoforms in BA24 to BA9 in depressed suicides (Figure 3b), 5 of the 21 isoforms displayed a difference greater than 20% from the median between the two cortical regions (B, AE, D, A and AC isoforms). Clear differences could be observed in the relative quantification of the proportion of 5-HT_2C_R mRNA isoforms between control and suicide groups when comparing both structures (Figure 3; compare panels a and b). Remarkably, the relative proportion of isoform D was higher in BA24 than in BA9 of non-psychiatric control subjects (*P*<0.001), whereas its relative proportion was lower in BA24 (*P*<0.05) of suicides (Figure 3b; compare isoform D between groups). In comparison with the non-psychiatric controls, in depressed suicides the relative proportion of the B isoform is even higher in BA24 compared with BA9 (Figure 3b). In addition, comparing 5-HT_2C_R-editing profiles in both brain areas of depressed suicides showed a significantly lower relative proportion of A isoform and a higher relative proportion of AC in BA9 (Figure 3b). Taken together, the data clearly show substantial differences in 5-HT_2C_R editing in the two cortical regions within non-psychiatric controls and depressed suicides.

### Specific suicide-induced changes in 5-HT_2C_R mRNA editing

Next, we analyzed the relative mRNA-editing profile of 5-HT_2C_R by focusing specifically on each cortical area in both non-psychiatric controls and depressed suicides (Figure 1; Q3 and Q4). When comparing the relative proportion of 5-HT_2C_R mRNA isoforms in non-psychiatric control subjects and depressed suicides in BA24 (Figure 3c), 3 of the 21 isoforms displayed a difference greater than 20% from the median between the two groups (D, A and ABDE isoforms). The relative proportion of A and ABDE most significantly increased in BA24 of depressed suicides, whereas that of the isoform D decreased. The relative proportions of the fully edited isoform ABCDE was also higher in BA24 of depressed suicides compared with non-psychiatric accidental controls. In BA24, the median value of the relative proportion of the infrequently edited isoform D was lower (−37% *P*<0.001) in depressed suicides (Figure 3c; upper bar D), whereas the relative proportion of frequently edited isoforms was higher as exemplified by ABCD, ACDE, ABC, ABCDE and ABDE isoforms. In general, the relative proportions of unfrequently edited isoforms (D, NE, AB, AD, CD and C) were lower in BA24 of suicides compared with that in accidental controls (Figure 3c). The relative proportion of the fully edited isoform (ABCDE) was higher both in BA9 and BA24 of depressed suicides, although the *P*-values were higher than *P*>0.05 (Figure 3c and d), likely reflecting a tendency. In BA9, only the relative proportion of AB was significantly higher in depressed suicides compared with non-psychiatric controls (Figure 3d). For both AE (*P*=0.01) and AC isoforms (*P*=0.07) the increase in the relative proportion of depressed suicides did not exceed 20% (Figure 3d). In the BA9 cortical region, a tendency to lower relative proportion of isoform ACE was found in suicides.

**Figure 4:**
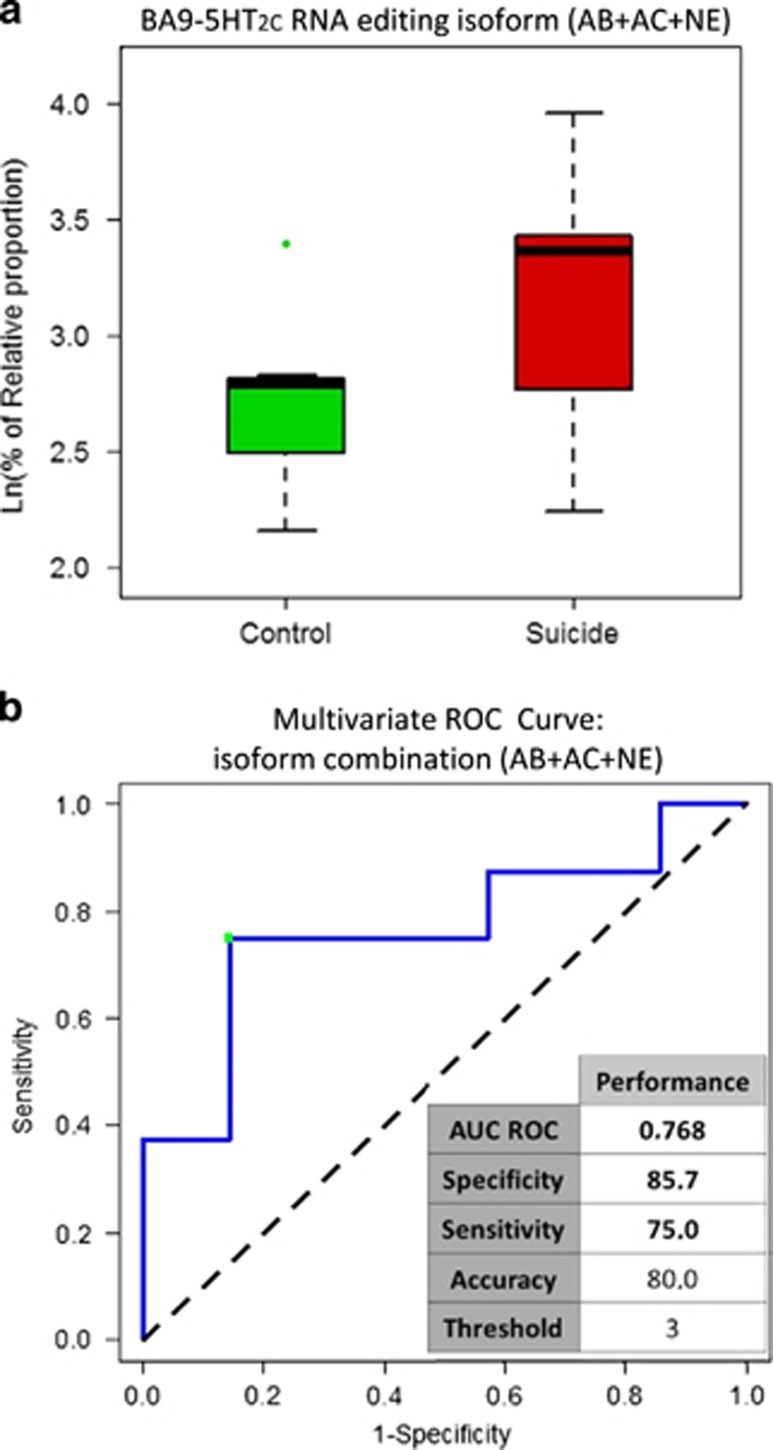
Suicide-induced alterations on the relative proportion of three isoforms of 5-HT_2C_R mRNA in Brodmann area 9 (BA9). (**a**) Sum of the relative proportion (%) of the AB, AC and NE 5-HT_2C_R isoforms in control (*n*=7) and suicide groups (*n*=8). (**b**) The combination of the AB-, AC- and non-edited (NE) isoforms was used to determine the threshold value and the performances of the diagnostic test by mROC. The performances are shown in the insets under the mROC curve.

**Figure 5:**
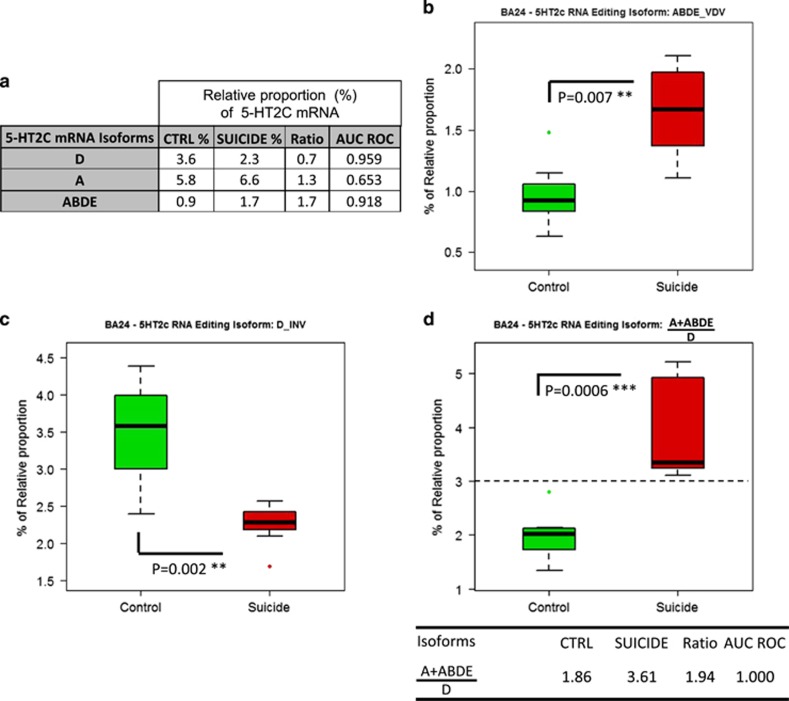
Suicide-induced alterations of the relative proportion of 5-HT_2C_R isoforms in BA24. (**a**) Table showing the most significant changes of the relative proportion of 5-HT_2C_R mRNA isoforms in BA24 of the suicide group (see Figure 4a). (**b, c**) Comparison of the relative proportion of ABDE and D isoforms in control (*n*=7) and suicide groups (*n*=7). Boxplot represents ln(x) of the relative proportion of 5-HT_2C_R mRNA in BA24 of both groups and s.e.m. (**d**) Sum of the relative proportion (%) of the non-edited (NE), A and ACD 5-HT_2C_R isoforms in control and suicide groups. **P⩽0.01 and ***P⩽0.001.

Next, we analyzed the relative proportion of a combination of isoforms in the DLPFCx (AB+AC+NE) and tested both specificity and sensibility by the mROC program (Figures 4a and b). Interestingly, the sensibility and specificity of this combination was high (Figure 4b). In the ACCx, the relative proportion of the three isoforms observed to be the most significantly altered between non-psychiatric controls and depressed suicides are shown in Figure 5a (ABDE, A and D isoforms). The isoform with the biggest change in relative proportion between control and suicide group is ABDE. The relative proportion of ABDE is increased by 70% in depressed suicides (Figure 5b), a significant alteration further underscored by the high discriminative value of the isoform (AUC ROC of 0.918). On the other hand, the relative proportion of isoform D was lowest in BA24 of suicides (Figure 5c). By examining the relative proportion of all isoforms that were most significantly altered in suicide, a combination of isoforms that could discriminate both groups in this study was identified (Figure 5d).

## Discussion

In the current study, using a powerful biochemical approach (CE-SSCP), we measured on post-mortem tissue the 5-HT_2C_R mRNA-editing profile in two distinct cortical areas of a small clinically well-defined cohort comprising non-psychiatric, drug-free control individuals and MDD patients who died by suicide. We observed marked differences in editing activity on 5-HT_2C_R mRNA in DLPFCx (BA9) and ACCx (BA24). Compared with non-psychiatric control individuals, clear alterations of editing levels of 5-HT_2C_R mRNA were detected in both cortical areas of depressed suicides. We further found that in the context of MDD patients who died by suicide substantially more changes of RNA-editing activity occur in the ACCx (BA24) compared with the DLPFCx (BA9), suggesting that the two closely related cortical regions adapt during pathology as two distinct functional modules. Quantitative PCR analysis of 5-HT_2C_R mRNA expression levels did not reveal significant differences between groups (Supplementary Figure S8). In BA24, the relative proportion of D isoform is most significantly decreased in suicides, whereas the ABDE isoform is most significantly increased, supporting the idea of an overall increase in RNA-editing activity in this area. The overall increase or RNA-editing activity is further underpinned by the trend toward a decrease in the NE and infrequently edited 5-HT_2C_R mRNA in BA24. Finally, by adding up the relative proportion of the most significant isoforms between non-psychiatric controls and depressed suicides, we present a 5-HT_2C_R mRNA ‘signature' that allows clear discrimination between the two groups (Figure 5d).

The methodological approach used in our study (CE-SSCP) generated from a single assay a comprehensive 5-HT_2C_R mRNA-editing profile of as many as 16 samples simultaneously. Linearity and reliability of this method was previously evaluated in the prefrontal cortex of the mouse.^28^ The traditionally used cloning–sequencing approach to determine 5-HT_2C_R mRNA-editing profile is known to introduce a bias because of the unavoidable sampling step of bacterial clones that are picked for sequencing. Another method widely used to evaluate RNA editing consisted of poisoned primer extension assays. This approach gives information of RNA editing at specific sites but cannot inform on the relative proportion of each individual mRNA isoform.^51^ Current CE-SSCP technology accurately detects the relative proportion of all 5-HT_2C_R isoforms within each sample. To our knowledge, apart from the RNA integrity number that evaluates ribosomal RNA integrity (Supplementary Figure S2), no means exist to qualitatively evaluate 5-HT_2C_R mRNA before amplification. However, its quality was best illustrated by the individual electrophoresis signals and the small variation of their mean over its 11 000 points, which characterized the analytical CE signal for each group of samples and from each cortical region (Supplementary Figure S4A). The best-fitting process of each individual signal gave precise results (means *r*^2^ at 0.9 in each group). As opposed to next-generation sequencing methods that require a particular bioinformatical analysis (demultiplexing barcodes, read alignment and finally generation of an RNA-editing measure) current CE-SSCP signal directly translates the relative proportion of each mRNA isoform in the sample. Yet, our data remarkably corroborated recently reported mRNA-editing profiles of 5-HT_2C_R determined by next-generation sequencing on comparable post-mortem tissue, that is, DLPFCx (BA9) of human brain specimens suffering from MDD disorder who committed suicide.^26^ As calibration of current CE-SSCP methodology was performed by running the PCR product obtained using plasmids containing in each individual 32 possible 5-HT_2C_R isoforms as a template, we indirectly provide evidence that inosine is read as guanosine by the RT enzyme as the RNA-editing profiles observed between next-generation sequencing and CE-SSCP approaches are highly similar.

A recent clinical study on a large cohort (*n*>450) of MDD patients and healthy controls found a significant association between MDD and interferon alpha/beta signaling pathways' gene expression in whole-blood cells.^52^ Given the remarkable clinical observation that a considerable proportion (up to 30%) of hepatitis C patients receiving interferon alpha-based immunotherapy for treatment rapidly develop depressive symptoms and/or full-blown major depression related to changes in serotonergic system;^53, 54, 55^ a link between elevated interferon and psychiatric disorders seems evident. Type I interferon regulates the activity of the human immune system and ADAR was found to be included in the signaling pathway genes with the strongest associations with MDD. It has furthermore been well characterized that gene expression of ADAR is transcriptionally regulated by the interferon signaling pathways,^18, 56, 57^ likely suggesting that the increase in ADAR expression observed in MDD patients, in part, could be a direct interferon-mediated effect. Moreover, a recent observation found an increase in ADAR1 gene expression in the DLPFCx (BA9) of exclusively MDD patients who committed suicide and not in that of patients diagnosed with bipolar disorder or schizophrenia.^58^ Interestingly, while a longstanding body of evidence links MDD to inflammation,^59^ accumulating data have recently highlighted the role of cytokines in suicidal behavior.^60, 61^ Among the many cytokines evaluated, altered levels of interleukin-1 beta and interleukin-6 were the most robust changes in cytokine levels associated with nonfatal suicide attempts that could discriminate suicide attempters from depressed patients.^62^

We previously analyzed, using our CE-SSCP technology, the changes induced on 5-HT_2C_R mRNA-editing levels by interferon treatment in SH-SY5Y human neuroblastoma cell line. This allowed us to predict the likelihood of a report to be emitted, or the Food and Drug Administration issuing an alert on medications for inducing depression and/or suicidal behavior.^56^ Analysis of the data with the mROC program allowed optimization of the combination of biomarkers in the context of the carefully selected cell line. Interestingly, by applying an optimized combination (AB+AC+NE) on the data obtained in the current study on human cortical tissue, we obtained a sensitivity of 75% and specificity of 85.7% (Figure 4), suggesting that similar changes in editing activities may exist in both studies. A common mechanism may underlie these adaptations in RNA-editing activities and speculatively may be linked to or associated with neuroinflammatory processes. A clear difference, however, is the limited ADAR2 activity in SH-SY5Y cells and accordingly reduced editing on the D and E sites in the cell line model.

Functional neuroimaging data have shown a dysregulation of limbic–cortical pathways in depression and treatment-resistant depression. The ACCx (BA24) has been proposed to be the critical link in the interaction of dorsal/cortical and ventral/limbic networks involved in the regulation of emotion.^30, 31^ Modulation of the ACCx (BA24) by repetitive transcranial magnetic stimulation has recently regained interest by psychosurgeons as an add-on procedure for treatment-resistant depressed patients. We supplement above-mentioned functional neuroimaging data and provide evidence for biochemical changes in the ACCx of depressed suicides. Recent data further suggested that cortical astrocytic function is influenced in the context of mood disorders and suicide in BA24, as evidenced by an increase in astrocytes in this specific region in post-mortem brains.^63^ Therefore, we cannot exclude that underlying changes in cell composition may also contribute to the observed alterations in RNA editing, although care was taken during tissue preparation to avoid confounds caused by cytoarchitectonic variations between the samples. Considering that the main risk factor for suicide is a mental health condition, it is therefore not possible to exclude the possibility that the observed changes are more related to MDD than to suicide. Yet, current data on RNA editing of 5-HT_2C_R further support the notion of a substantial change in steady-state RNA-editing activity in the ACCx of MDD patients who committed suicide, likely reflecting underlying changes in global functioning of this area as determined by functional neuroimaging.

Recently, experience-dependent changes in RNA editing of the glutamate receptor in amygdala and hippocampus have been reported in mice, further supporting the idea that region-specific alterations of RNA-editing activity modulate synaptic function and processes.^64^ In the current study we found significant alterations of RNA-editing processes in two functionally distinct neocortical areas in post-mortem brains of depressed suicide victims. We describe a specific RNA-editing ‘signature' and propose its use to predict the probability of drugs to trigger similar alterations by analysis of the A-to-I editing of specific targets at the periphery (for example, T cells in the blood). In turn, this would open the way to predictive RNA-editing-based blood biomarkers of the risk to develop depressive symptoms and suicide attempt. Altogether, an increasing body of evidence converges to the hypothesis that interferon is closely implicated in the biology of MDD and/or suicide, tentatively linking altered RNA-editing activities in cortical areas and suicide neurobiology.

## Figures and Tables

**Table 1:** Baseline characteristics of the study population

*Main population characteristics*	*Control (*n=*8)*	*MDD (*n=*8)*	*Control versus MDD* P*-value*
*Age (years)*			
Mean	37.4 (6.5)	38.1 (6.5)	0.878
Min–max	16–60	14–62	
			
Sex (male), *n* (%)	8 (100.0)	8 (100.0)	NA
			
*Ethnicity*, n *(%)*			
White	4 (50.0)	5 (62.5)	0.486
African American	3 (37.5)	1 (12.5)	
Hispanic	1 (12.5)	2 (25.0)	
			
*Axis I*, n *(%)*			
MDD	0 (0.0)	8 (100.0)	NA
None	8 (100.0)	0 (0.0)	
			
*Toxicology/treatments*, n *(%)*			
CO	1 (12.5)	0 (0.0)	0.262
Analgesics	0 (0.0)	2 (25.0)	
Anesthetics	1 (12.5)	0 (0.0)	
None	6 (75.0)	6 (75.0)	

Abbreviations: CO, carbon monoxide; MDD, major depressive disorder.

Data are expressed as mean value (s.e.m.). Statistical analysis was performed using Wilcoxon rank-sum test (*P*-values of main characteristics are shown). Statistical analyses of ethnicity, Axis I and toxicology were obtained using *X*^2^-test.

**Table 2:** Baseline characteristics of tissue specimen

*Tissue characteristics*	*Control (*n=*8)*	*MDD (*n=*8)*	*Control versus MDD* P*-value*
Weight (mg)	78.7 (8.6)	79.4 (4.8)	0.328
pH	6.6 (0.05)	6.7 (0.05)	0.382
RNA integrity number (RIN)	7.7 (0.2)	7.7 (0.2)	0.908
Post-mortem interval (hours)	13.1 (2.3)	18.2 (2.2)	0.161

Abbraviations: MDD, major depressive disorder; RIN, RNA integrity number.

Data represent mean value (s.e.m.) and statistical analysis were performed using Wilcoxon rank-sum test (*P*-values of main characteristics are shown).
