# Supplementary material for: Region-specific alterations of A-to-I RNA editing of serotonin 2c receptor in the cortex of suicides with major depression
Source: Transl Psychiatry. 2016 Aug 30;6(8):e878–. doi: 10.1038/tp.2016.121 (PMC5022077; doi:10.1038/tp.2016.121)
Supplement: Supplementary Information [file tp2016121x1.doc]

**SUPPLEMENTAL DATA**

**REGION-SPECIFIC ALTERATIONS OF A-TO-I RNA EDITING OF SEROTONIN 2C RECEPTOR IN THE CORTEX OF SUICIDES WITH MAJOR DEPRESSION**

Dinah Weissmann, PharmD, PhD

Siem van der Laan, PhD

Mark D. Underwood, PhD

Nicolas Salvetat, MSc

Laurent Cavarec, PhD

Laurent Vincent, MSc

Franck Molina, PhD

J. John Mann, MD

Victoria Arango, PhD

Jean François Pujol, MD, PhD

**Supplemental Figure legends**

**Table S1** Detailed characteristics of the study population.

**Figure S1.** (A) Coronal section at a level just posterior to the genu of the corpus callosum, (*; red asteriks) indicates Brodmann Area 9, as identified by cytoarchitectonic features provided by the Nissl-stain section in (B), which shows how BA9 differs from near adjacent BA46 (with a BA9/BA46 transition area in between) by lacking a well-developed Layer IV. (C). A coronal section, just posterior to 1A, (*; red asteriks) indicating the block of anterior cingulate cortex from where the punches were taken. In (D), a section was stained for NeuN in order to visualize cytoarchitectonic features.

**Figure S2.** (A) RNA integrity number (RIN) of total RNA extracted from brain tissue. With regard to the RIN numbers, except one, all samples yielded a score above 6, indicative of sufficient RNA quality. (B) Representative gel-like image of obtained electrophoregram by analysis on the Bioanalyzer.

**Figure S3.** Schematic representation of the Capillary Electrophoresis – Single Stranded Conformational Polymorphism (CE-SSCP) procedure. A first target amplification is performed, followed by a nested PCR to fluorescently label the cDNA. Next, the samples are denaturated by heating and kept on ice prior analysis on the Genetic Analyzer.

**Figure S4.** (A) Mean capillary electrophoretic (CE) signal obtained in the DPFCx of the control group is represented by the black plain curve. The red dotted curve corresponds to this mean incremented on each point of the function by its corresponding SEM (n=7). After negative transposition, some typical curves identified the typical CE signal of standards corresponding to NE, A, AB, AE, ABCDE, ABD, BC, ABCD, B and C edited isoforms of the 5-HT2CR R mRNA isoforms. The time basis is measured in scans (there are 6,22 scans/second). For the combined analysis of the signal obtained with the two fluorescent tags (VIC and Fam) the signals are set on the same time basis, normalized to zero for VIC-labeled strand signal and normalized to 5000 for Fam-labeled one. When an isoform could not be resolved with one labeled strand, it could be resolved with the other (see shaded cells). In the inlet a representative situation of signal amplification to allow correct separation of the isoforms NE and C. The non-edited (NE) and each 32 isoforms used as calibration standard are characterized by the migration time of its principal peak (B). Two peaks could be considered as separate when the delta T between their respective migration times was ≥ 20 scans.

**Figure S5.** Statistical analysis of 5-HT_2C_R mRNA editing profile in BA9 and BA24 of control and suicide victims. (A) Three different statistical approaches were employed to test the data set. Dispersion trees, Pearson correlation matrix and principle component analysis all identified the three same samples as distant. (B) Most distant data set were excluded from the analysis resulting in more homogeneous data.

**Figure S6.** Complete raw data set (n=8) of 5-HT_2C_R mRNA editing profile in BA24 in control (A) and Suicide (B) male subjects. The individuals that were identified as distant by the biostatistical analysis were excluded and marked in red.

**Figure S7.** Complete raw data set (n=8) of 5-HT_2C_R mRNA editing profile in BA9 in control (A) and Suicide (B) male subjects. The individuals that were identified as distant by the biostatistical analysis were excluded and marked in red.

**Figure S8.** qPCR analysis of 5-HT_2C_R in both cortical areas. Data shows the standard curve for each assay. Data is given as Ct and ration between the groups (controls and suicides) in BA9 (p=0.962) and BA24 (p=0.244) is shown. No difference in -HT_2C_R mRNA expression could be observed.

**Supplemental Material and methods**

**RNA isolation, cDNA synthesis and PCR**

Total RNA was extracted from brain specimens, purified (Qiagen RNeasy, Kit), quantified by spectrophotometry, treated with 1 unit of DNase I (Invitrogen) for 15 min at room temperature in a final volume of 10µl, then 1µl of 25mM EDTA was added and the mixture heated for 10 min at 65°C. Next, total RNA was qualified by electrophoresis and the RNA integrity number (RIN) score was determined for each total RNA sample (Fig. S2). Reverse transcription was performed using 15 units of ThermoScript reverse transcriptase (ThermoScript RT-PCR System, Invitrogen) in presence of Oligo(dT) primers at a final concentration of 2.5µM**.**  Prior analysis of 5-HT_2C_R receptor RNA editing levels, non-denaturating capillary electrophoresis-single stranded conformational polymorphism (CE-SSCP) procedure was calibrated using plasmid containing exactly a 250bp long cDNA sequence coding respectively for all 32 possible edited isoforms (Figure S3 and S4). All expression plasmids were verified by DNA sequencing. An initial amplification by PCR (final volume 25µl) was carried out on 1µl of the obtained cDNA using 0.2 unit of Platinum *Taq* DNA polymerase (ThermoScript RT-PCR system, Invitrogen) and specific intron-spanning 5-HT_2C_R primers (forward primer: 5’-TGTCCCTAGCCATTGCTGATATGC-3’ and reverse primer: 5’-GCAATCTTCATGATGGCCTTAGTC-3’; final concentration of each 0.2µM) located on exon IV and exon V, respectively. The PCR protocol consisted of an initial denaturing step at 95°C for 3 min, 35 cycles of amplification (15s at 95°C; 30s at 60°C; 20s at 72°C), and a final elongation step of 2 min at 72°C. One µl of a 1/50 dilution of the PCR products or the 250 bp cDNA amplified from plasmids containing the 32 standards of human 5-HT2CR R isoforms, were used as templates for nested-PCR. The second amplification was performed in a final volume of 20µl with VIC and FAM fluorescent primers. The primer sequences used were: Forward: 5’-ATGTGCTATTTTCAACAGCGTCCATC-3’ and Reverse: 5’-GCAATCTTCATGATGGCCTTA-3’. This set of primer pair was optimised for conformational analysis of human 5-HT_2C_R mRNA editing by non-denaturing CE-SSCP. The length of the amplified fragment was carefully chosen (127 bp). Quality of the amplified fragments was assessed on a 2% agarose gel before subsequent analysis in a 3100 Avant Genetic Analyser (Applied Biosystem).

**Quantitative real-time PCR (qPCR) of the 5-HT2cR mRNA**

Total RNA (1µg) from samples was reverse transcribed with the ThermoScript RT-PCR System (Invitrogen) using oligo-dT priming. Relative mRNA expression of 5-HT2cR (HTR2C) was measured by qPCR using the StepOnePlus Real-Time PCR System (Applied Biosystems) and a gene-specific TaqMan FAM/MGB assay (Hs 00968671_m1, Applied Biosystems). For each sample, a 1:3 dilution of the reverse transcription product was amplified and run in triplicates. The relative expression level of the target in suicides versus controls was calculated using the relative standard curve method according to Applied Biosystems instructions (Guide to Performing Relative Quantitation of Gene Expression Using Real-Time Quantitative PCR). An independent sample (Human Brain, cerebral cortex total RNA, Clontech) was chosen for the standard curves construction. Starting from 1µg of RNA, a serial dilution (1 :3) of the cerebral cortex total RNA was performed up to 12.3 or 4.1 ng. Three independent reverse transcription reactions were carried out for each dilution, amplified and assayed.

**Statistical analysis and design of the analysis**

The data quality evaluation was performed by three distinct approaches :

*Dispersion tree.* The dispersion tree method, as described in [ref], was used. Briefly, editing values in each experiment were represented by an expression vector of dimension *n* (n being the number of editing isoforms). The Euclidian distance between vectors, which represents all experiments and their editing values, was calculated. The resulting distance matrix was used to perform a clustering of all the experiments. The clustering and the resulting unrooted “dispersion tree” graphical representation were performed with the PHYLIP v3.6 package (<http://evolution.genetics.washington.edu/phylip.html>).

*Pearson correlation matrix****.*** The Pearson correlation coefficient measures the strength of the linear relationship between two experiments. For x and y *experiments*, it is denoted as *r* and computed as:

$$r_{xy}=\frac{\sum_{i=1}^{n} \left(x_{i}-\bar{x}\right)\left(y_{i}-\bar{y}\right)}{\sqrt{\sum_{i=1}^{n} \left(x_{i}-\bar{x}\right)^{2}\sum_{i=1}^{n} \left(y_{i}-\bar{y}\right)^{2}}}$$

If there is an exact linear relationship between two experiments, the correlation is 1 or –1, depending on whether the variables are positively or negatively related. If there is no linear relationship, the correlation is close to zero. A matrix of correlation coefficients is calculated and a colour map of the Pearson correlations coefficients is plotted to show the groups of experiments that have similar correlations (on a scale from red (+1) to green (-1)) (Figure S6A-B).

*Principal component analysis (PCA).* PCA is a technique to take linear combinations of the original variables such that the first principal component has maximum variation; the second principal component has the next most variation subject to being orthogonal to the first and the remaining components must all display these two features. The ranking of components in decreasing order determines the data variability. PCA is thus used to attribute the overall data variability to a reduced set of variables, which are called “principal components”. The first two principal components are used to map each experiment in a 2D plot. With this approach, an experiment that deviates too much from the others can be considered as an outlier (Figure S6A).
